# Projected lifetime cancer risks from occupational radiation exposure among diagnostic medical radiation workers in South Korea

**DOI:** 10.1186/s12885-018-5107-x

**Published:** 2018-12-04

**Authors:** Won Jin Lee, Yeongchull Choi, Seulki Ko, Eun Shil Cha, Jaeyoung Kim, Young Min Kim, Kyoung Ae Kong, Songwon Seo, Ye Jin Bang, Yae Won Ha

**Affiliations:** 1 Department of Preventive Medicine, Korea University College of Medicine, 73, Inchon-ro, Seongbuk-gu, Seoul, 02841 South Korea; 2 Seoul Workers’ Health Center, Ewha Womans University, Seoul, South Korea; 3 Department of Preventive Medicine, Keimyung University School of Medicine, Daegu, South Korea; 4 Department of Statistics, Kyungpook National University, Daegu, South Korea; 5 Department of Preventive Medicine, College of Medicine, Ewha Womans University, Seoul, South Korea; 6 Laboratory of Low Dose Risk Assessment, National Radiation Emergency Medical Center, Korea Institute of Radiological and Medical Sciences, Seoul, South Korea

**Keywords:** Medical radiation workers, Neoplasms, Radiation exposure, Risk assessment

## Abstract

**Background:**

Timely assessment of cancer risk from current radiation exposure among medical radiation workers can contribute to the development of strategies to prevent excessive occupational radiation exposure. The purpose of the present study is to estimate the lifetime risk of cancers induced by occupational radiation exposure among medical radiation workers.

**Methods:**

Using estimated organ doses and the RadRAT risk assessment tool, the lifetime cancer risk was estimated among medical radiation workers who were enrolled in the Korean National Dose Registry from 1996 to 2011. Median doses were used for estimating the risk because of the skewed distribution of radiation doses. Realistic representative exposure scenarios in the study population based on sex, job start year, and occupational group were created for calculating the lifetime attributable risk (LAR) and lifetime fractional risk (LFR).

**Results:**

The mean estimated lifetime cancer risk from occupational radiation exposure varied significantly by sex and occupational group. The highest LAR was observed in male and female radiologic technologists who started work in 1991 (264.4/100,000 and 391.2/100,000, respectively). Female workers had a higher risk of radiation-related excess cancer, although they were exposed to lower radiation doses than male workers. The higher LAR among women was attributable primarily to excess breast and thyroid cancer risks. LARs among men were higher than women in most other cancer sites. With respect to organ sites, LAR of colon cancer (44.3/100,000) was the highest in male radiologic technologists, whereas LAR of thyroid cancer (222.0/100,000) was the highest in female radiologic technologists among workers who started radiologic practice in 1991. Thyroid and bladder cancers had the highest LFR among radiologic technologists.

**Conclusions:**

Our findings provide an assessment of the potential cancer risk from occupational radiation exposure among medical radiation workers, based on current knowledge about radiation risk. Although the radiation-related risk was small in most cases, it varied widely by sex and occupational group, and the risk would be underestimated due to the use of median, rather than mean, doses. Therefore, careful monitoring is necessary to optimize radiation doses and protect medical radiation workers from potential health risks, particularly female radiologic technologists.

**Electronic supplementary material:**

The online version of this article (10.1186/s12885-018-5107-x) contains supplementary material, which is available to authorized users.

## Background

Medical radiation workers represent the largest group of workers who are occupationally exposed to radiation, and their number is rapidly increasing worldwide [1]. These workers constitute a well-defined population with a certificate of professional qualification, and they include a large proportion of female workers compared with other occupations; this increases the importance of investigating radiation exposure and sex-specific health effects. Although the radiation exposure levels in medical radiation workers are usually low, these workers include high-exposure subgroups, such as those performing fluoroscopically guided procedures, and with long-term, chronic low dose-rate exposures, which may correspond to the type of exposure in the general population. Considering these characteristics, a few cohort studies have been conducted among medical radiation workers to date [2, 3]. Previous epidemiological studies reported an increase in the risk of cancer among workers employed in early years (e.g., 1950s), indicating the need to assess lifetime cancer risk in medical radiation workers based on current radiation exposure levels [3]. Moreover, information on the potential long-term cancer risk considering current exposure levels is helpful to optimize work procedures and develop strategies to prevent excessive occupational radiation exposure.

Risk projection methods can estimate the excess risk of cancer from radiation exposure in populations [1, 4, 5]. These methods have been implemented to assess cancer risk from medical radiation [6–8], radioactive fallout from nuclear weapons testing [9], radon exposure [10], Chernobyl [11] and Fukushima accidents [12]. However, for medical radiation workers, to date, only few studies have estimated lifetime attributable risk (LAR) of occupational exposure in cardiologists in Italy [13] and vascular surgeons and scrub nurses in Korea [14]. In addition, these assessments were based on the simple application of radiation doses to summary tables in the Biological Effects of Ionizing Radiation VII (BEIR VII) report [15]. However, other factors such as organ susceptibility to radiation, sex, age at exposure, and baseline incidence rates significantly affect the risk of developing radiation-related cancers.

The number of medical radiation workers increased significantly in South Korea, from 12,652 in 1996 to 80,115 in 2016 [16]. A historical dose reconstruction was conducted, and organ-specific radiation doses were estimated for all diagnostic medical radiation workers who were enrolled in the National Dosimetry Registry (NDR) from 1996 to 2011 as part of the registry-linkage cohort study [17]. Using these organ-specific doses to estimate cancer risk could provide valuable information of cancer burden from current levels of occupational radiation exposure among medical radiation workers. This approach is particularly beneficial when the follow-up of cohorts is short and cohort members are relatively young.

Therefore, the purpose of this study was to estimate the lifetime cancer risk from occupational radiation exposure and determine differences in cancer risk by sex and occupational group among Korean medical radiation workers. Considering the rapid increase in the number of medical radiation workers and medical radiation practices worldwide, these findings will help provide a timely assessment of cancer risk based on current radiation exposure levels and identify priority groups for radiation protection among medical radiation workers.

## Methods

### Study population and estimation of organ doses

The study population comprised 94,396 diagnostic radiation workers who enrolled in the NDR from 1996 to 2011, including radiologists (*n* = 1520), other physicians (*n* = 18,684), dentists (n = 15,705), dental hygienists (*n* = 13,488), radiologic technologists (*n* = 26,356), nurses (*n* = 7561), and other medical assistants (*n* = 11,082). The Korea Center for Disease Control and Prevention (KCDC) has been conducting monitoring programs for all radiologic technologists (both conventional and interventional) involved in diagnostic radiology since 1996. In addition, the KCDC maintains a centralized national dose registry and operates a lifelong follow-up management system for radiation dose [16]. Medical workers involved with nuclear medicine and therapeutic departments are under the Nuclear Safety and Security Commission and were not included in this system. Registry information included name, sex, personal identification number, occupational group, quarterly dose data, and the beginning and end of the period of measurement. Dose measurements were collected quarterly by five personnel monitoring centers designated by the KCDC using a personal thermoluminescent dosimeter. Standard practice is for dosimeters to be worn under aprons at chest level. All instruments were calibrated annually.

Organ-specific doses were previously estimated for all diagnostic radiation workers after badge doses were calculated for workers exposed before 1996 [17]. Briefly, the annual and cumulative individual badge doses based on Hp (10) (dose at a tissue depth of 10 mm from the dosimeter) were calculated by combining the quarterly badge readings for the workers enrolled in the NDR. Quarterly doses below 0.01 mSv, which is the lowest detectable level of NDR, were assigned at value of 0.005 mSv – the midpoint between 0.01 mSv and zero at the dose reconstruction. For workers who started working with radiation before 1996 (*n* = 13,178; 14.0% of the total enrollees in the NDR), historical badge doses were reconstructed using a model in which annual doses were determined as a log-linear function of time and age [18]. The age at the time of first exposure was estimated for each sex and occupational group using the findings of our previous survey [19] to determine the first year of radiologic practice for individual workers. Then, organ doses were estimated by converting measured and reconstructed individual badge doses to each organ-specific dose and multiplying by two conversion coefficients provided by the International Commission on Radiological Protection: the organ-absorbed dose per unit of air kerma free-in-air and the personal dose equivalent per unit of air kerma free-in air [20, 21]. The organ dose methods was adjusted for the probability of apron use and the placement of the badge, and an attenuation rate of 0.8 was assumed for the use of a lead apron to reflect the shielding effect. The organs and tissues for which specific doses were estimated included the bladder, brain/central nervous system, breast, colon, esophagus, gallbladder, kidney, liver, lung, oral cavity and pharynx, ovary, pancreas, prostate, stomach, rectum, red bone marrow, thyroid, and uterus. The remainder included all solid cancers other than these solid tumors, excluding non-melanoma skin cancer.

### Exposure scenarios

For estimating realistic exposure scenarios of cancer risk, all workers were classified by sex, job start year, and occupational group. The estimated radiation doses were allocated to each group by year based on i) the reported NDR doses for the period of 1996–2011, ii) the reconstructed doses for the period before 1996, and iii) the 2011 NDR doses for the period after 2011 until 60 years of age based on the assumption that future radiation doses were the same dose as those in 2011. The age of 60 years was considered the average retirement age in South Korea (https://goo.gl/dtstFk), although the retirement ages varied slightly according to the occupational group. Radiation organ doses had right-skewed distribution; thus they were represented using two parameters of lognormal distribution - median and geometric standard deviations - to incorporate the dose uncertainty. After that, the age of first exposure according to sex and occupational group was imputed by accessing data from our previous survey on the average age of start of radiologic practice by diagnostic medical radiation workers [19]. Three calendar years (1991, 2001, and 2011) of start of professional practice were selected to cover the full range of exposure scenarios in this population. Furthermore, 42 scenarios combining both sexes, three distinct years of start of professional practice, and seven occupational groups were constructed using NDR and survey data.

### Prediction of cancer risk

Excess lifetime cancer risk was estimated for each scenario using the RadRAT risk assessment tool (RadRAT version 4.1.1), which was developed at the United States National Cancer Institute to estimate the lifetime risk of developing cancer from radiation exposure [22]. The RadRAT program was developed based on the BEIR VII models [15] to estimate excess cancer risk from radiation exposure by incorporating current understanding about radiation-related risks with an allowance for uncertainties related to dose-response model parameters, minimum latency periods, dose and dose-rate effectiveness factor, and risk transport from population to population. The RadRAT program follows the BEIR VII assumptions by applying an uncertain dose and dose-rate effectiveness factor (DDREF) for all chronic exposures using a lognormal distribution with a geometric mean of 1.5 with no-threshold risk models. Input information required by RadRAT includes sex, year of birth, exposure history, and run-specific parameters [22]. The RadRAT program can be freely accessed at https://irep.nci.nih.gov/radrat and has been applied in previous radiation-related cancer risk projection studies [7, 23–25]. However, to our knowledge, no study to date has applied the RadRAT program to medical radiation workers. The lifetime attributable risk (LAR), the probability of a premature incidence of cancer attributable to radiation exposure in a representative member of the population (i.e., the probability that an exposed population will develop a radiation-induced cancer during their lifetime), was calculated as excess cases per 100,000 by sex, occupational group, and first year of professional practice in this program. The lifetime baseline risk (LBR) for cancer, the cumulative baseline probability of having a cancer over the lifetime, was calculated based on South Korean cancer incidence rates in 2010. The lifetime fractional risk (LFR) – the ratio between LAR and LBR – was presented to express attributable risk relative to baseline risk; LFR is more stable than LAR regarding differences in population structure and cancer incidence rates [26].

The 42 exposure scenarios were applied to individual organs, 15 for male and 17 for female workers, producing a total of 672 lifetime exposure scenarios (i.e., seven occupational groups × three distinct years of start of professional practice × 15 organ sites for men plus seven occupational groups × three distinct years of start of professional practice × 17 organ sites for women). For each scenario, we input data on sex, year of birth calculated as the first year of work minus the age at the first year of work, and each organ dose considered as chronic exposure in the RadRAT program. Sex- and age-specific incidence rates in South Korea in 2010 were used for determining the baseline incidence rates and survival function was based on US general population 2000–2005 in the RadRAT program. Organ-specific lifetime cancer risk was estimated in each of the 672 exposure scenarios using the organ-specific median annual absorbed doses per job title at the specified age of first exposure beginning in three separate calendar years of work start (1991, 2001, and 2011) and ending at an assumed retirement at age 60 years. The LAR of all cancers combined was calculated as the sum of all risks of individual organs. The leukemia risk was estimated excluding chronic lymphocytic leukemia. The excess cancer risk was calculated with 90% uncertainty intervals to incorporate both statistical and subjective uncertainties computed by Monte Carlo simulations using the RadRAT program and the simulation sample size was 300. An example of the risk estimation process can be found in the Additional file 1: Figure S1.

## Results

Table 1 shows the information used for estimating the lifetime cancer risk, including sex, occupational groups, and three distinct years of beginning radiologic practice. Female and male workers started radiologic practice at the age of 21–29 years and 25–35 years, respectively, demonstrating that total exposure duration was longer for female workers than for male workers for all occupational groups, assuming a retirement age of 60 years. The median cumulative radiation doses were higher in male workers than in female workers in most cases. Male radiologic technologists had the highest median doses when they started working in 1991, whereas dentists had the lowest doses. The temporal decrease in the median cumulative radiation dose by year started work varied by occupational group, and radiologic technologists and nurses tended to have the lowest relative decrease. The dose distribution was wide by sex, job title, and calendar years.

**Table 1:** Occupational characteristics of diagnostic medical radiation workers by occupational group and sex

Occupational group	Sex	Number	Age at first exposure (mean)	Exposure duration (mean, years)	Cumulative badge doses by job start year (median, mSv)		
					1991	2001	2011
Radiologic technologist	Male	17,278	25	36	22.86	11.72	9.18
	Female	9078	22	39	10.85	6.92	5.66
Radiologist	Male	1057	26	35	10.99	3.15	1.93
	Female	463	26	35	12.33	3.22	1.38
Other physician	Male	15,986	35	26	4.36	1.65	0.78
	Female	2698	29	32	5.21	2.48	1.60
Dentist	Male	12,279	25	36	3.01	1.77	1.26
	Female	3426	26	35	2.80	1.65	1.23
Dental hygienist	Male	70	26	35	4.45	3.26	3.33
	Female	13,418	22	39	3.14	2.08	1.76
Nurse	Male	424	27	34	12.68	4.89	3.74
	Female	7137	22	39	10.55	3.97	3.32
Other	Male	6776	25	36	6.53	3.30	2.88
	Female	4306	21	40	4.40	2.78	2.20

The LAR and LFR for all cancers combined by occupational group and year of beginning radiologic practice in male workers are summarized in Table 2. There were considerable variations in the estimated cancer risk between the occupational groups, and the highest LAR was observed in radiologic technologists (215.4/100,000–264.4/100,000) whereas the lowest LAR was found in dentists. A tendency of temporal decrease in LAR and LFR was detected by year of beginning professional practice in all occupational categories and was more prominent between 1991 and 2001 than in 2011. The comparison of LARs to baseline risks indicated that radiologic technologists had the highest LFR of all cancers combined (0.43–0.52%), whereas dentists had the lowest LFR.

**Table 2:** Lifetime cancer risks by occupational group and year of beginning radiologic practice among male diagnostic medical radiation workers

Occupational group	Job start year	LAR	LFR (%)	LBR
		(90% UI)	(90% UI)	
Radiologic technologist	1991	264.4 (60.6–667.3)	0.52 (0.12–1.32)	
	2001	243.4 (45.4–631.5)	0.48 (0.09–1.25)	
	2011	215.4 (38.1–569.6)	0.43 (0.08–1.13)	50,496
Radiologist	1991	83.7 (22.9–208.3)	0.17 (0.05–0.41)	
	2001	38.2 (8.6–96.2)	0.08 (0.02–0.19)	
	2011	21.6 (5.4–54.6)	0.04 (0.01–0.11)	50,497
Other physician	1991	35.3 (8.3–95.1)	0.07 (0.02–0.19)	
	2001	18.9 (4.2–54.6)	0.04 (0.01–0.11)	
	2011	8.8 (2.2–23.0)	0.02 (0.00–0.05)	50,381
Dentist	1991	24.1 (6.1–61.1)	0.05 (0.01–0.12)	
	2001	17.0 (3.8–42.3)	0.03 (0.01–0.08)	
	2011	10.0 (2.7–23.4)	0.02 (0.01–0.05)	50,496
Dental hygienist	1991	133.0 (9.6–219.8)	0.26 (0.02–0.44)	
	2001	29.5 (7.7–73.5)	0.06 (0.02–0.15)	
	2011	28.9 (7.8–70.4)	0.06 (0.02–0.14)	50,497
Nurse	1991	158.8 (32.7–434.0)	0.31 (0.06–0.86)	
	2001	90.3 (17.0–246.6)	0.18 (0.03–0.49)	
	2011	64.1 (12.5–177.0)	0.13 (0.02–0.35)	50,493
Other	1991	80.8 (16.7–221.2)	0.16 (0.03–0.44)	
	2001	80.8 (16.7–221.2)	0.16 (0.03–0.44)	
	2011	39.9 (8.5–98.5)	0.08 (0.02–0.20)	50,496

*LAR* lifetime attributable risk (excess cases per 100,000, 90% uncertainty interval [UI]), *LFR* lifetime fractional risk (%, 90 uncertainty interval), *LBR* lifetime baseline risk (cases per 100,000)

The pattern of projected cancer risk by job title in female workers was similar to that of male workers (Table 3). The LAR in female workers was, however, higher than that in male workers for all occupational groups. Female radiologic technologists (322.1/100,000–391.2/100,000) and nurses (129.9/100,000–402.6/100,000) showed the highest LAR values whereas dentists had the lowest LAR. The tendency of temporal decrease in lifetime attributable cancer risk was greater in female workers than male workers, and the proportional reduction by year of start of professional practice was smaller in radiologic technologists than in other groups. The LFR for all cancers combined in female workers was higher than that in male workers for all occupational categories, and the differences in the LFR between the occupational groups in women were similar to those in men.

**Table 3:** Lifetime cancer risks by occupational group and year of beginning radiologic practice among female diagnostic medical radiation workers

Occupational group	Job start year	LAR	LFR (%)	LBR
		(90% UI)	(90% UI)	
Radiologic technologist	1991	391.2 (93.6–1006.8)	1.00 (0.24–2.58)	
	2001	382.6 (74.3–1078.6)	0.98 (0.19–2.77)	
	2011	322.1 (58.7–923.3)	0.83 (0.15–2.37)	38,991
Radiologist	1991	270.5 (67.7–701.5)	0.70 (0.17–1.81)	
	2001	100.7 (19.1–273.7)	0.26 (0.05–0.71)	
	2011	41.5 (10.0–107.5)	0.11 (0.03–0.28)	38,792
Other physician	1991	131.7 (28.7–361.8)	0.34 (0.07–0.94)	
	2001	79.7 (14.9–247.4)	0.21 (0.04–0.64)	
	2011	36.6 (8.3–95.8)	0.10 (0.02–0.25)	38,508
Dentist	1991	66.1 (14.7–174.1)	0.17 (0.04–0.45)	
	2001	48.3 (9.3–124.2)	0.12 (0.02–0.32)	
	2011	23.5 (6.0–58.8)	0.06 (0.02–0.15)	38,792
Dental hygienist	1991	105.3 (24.0–277.9)	0.27 (0.06–0.71)	
	2001	80.6 (16.5–240.3)	0.21 (0.04–0.62)	
	2011	48.0 (11.9–125.2)	0.12 (0.03–0.32)	38,991
Nurse	1991	402.6 (98.2–1090.1)	1.03 (0.25–2.80)	
	2001	222.8 (39.6–674.3)	0.57 (0.10–1.73)	
	2011	129.9 (28.1–350.3)	0.33 (0.07–0.90)	38,991
Other	1991	198.3 (40.7–787.3)	0.51 (0.10–2.02)	
	2001	153.5 (28.2–464.3)	0.39 (0.07–1.19)	
	2011	95.1 (20.1–255.6)	0.24 (0.05–0.65)	39,028

*LAR* lifetime attributable risk (excess cases per 100,000, 90% uncertainty interval [UI]), *LFR* lifetime fractional risk (%, 90 uncertainty interval), *LBR* lifetime baseline risk (cases per 100,000)

The organ-specific estimated excess cancer risks by sex and occupational group when professional practices started in 1991 is shown in Table 4. The LAR of colon cancer (44.3/100,000), stomach cancer (40.0/100.000), and lung cancer (36.1/100,000) was higher in male radiologic technologists, whereas the LAR of thyroid cancer (222.0/100,000) was higher in female radiologic technologists. The LFR of thyroid and bladder cancer was higher in both men (2.23 and 1.01%, respectively) and women (2.57 and 2.64%, respectively) than those of other sites.

**Table 4:** Lifetime cancer risks of selected cancer sites by sex among diagnostic medical radiation workers who started job in 1991

Organ sites	Male		Female	
	LAR	LFR (%)	LAR	LFR (%)
Bladder				
Radiologic technologist	18.0	1.01	12.0	2.64
Radiologist	5.2	0.29	7.0	1.54
Other physician	2.6	0.15	5.1	1.11
Dentist	1.6	0.09	2.1	0.46
Dental hygienist	8.1	0.45	2.8	0.62
Nurse	10.3	0.58	9.4	2.08
Other	5.3	0.30	5.1	1.11
Brain				
Radiologic technologist	1.3	0.39	0.2	0.07
Radiologist	0.4	0.13	0.1	0.04
Other physician	0.2	0.05	0.1	0.03
Dentist	0.1	0.04	0.04	0.01
Dental hygienist	0.6	0.19	0.1	0.02
Nurse	0.8	0.25	0.2	0.06
Other	0.4	0.12	0.1	0.03
Breast				
Radiologic technologist	–	–	50.5	1.15
Radiologist	–	–	35.7	0.82
Other physician	–	–	18.2	0.42
Dentist	–	–	8.8	0.20
Dental hygienist	–	–	13.5	0.31
Nurse	–	–	49.8	1.14
Other	–	–	24.6	0.56
Colon				
Radiologic technologist	44.3	0.99	13.9	0.44
Radiologist	13.3	0.30	8.4	0.27
Other physician	6.4	0.14	5.9	0.19
Dentist	3.9	0.09	2.4	0.08
Dental hygienist	23.2	0.52	3.3	0.10
Nurse	26.6	0.59	11.2	0.35
Other	13.1	0.29	6.0	0.19
Esophagus				
Radiologic technologist	6.3	0.55	0.8	0.68
Radiologist	2.0	0.17	0.5	0.42
Other physician	1.0	0.09	0.3	0.27
Dentist	0.6	0.05	0.1	0.12
Dental hygienist	3.0	0.26	0.2	0.16
Nurse	3.5	0.31	0.7	0.55
Other	1.8	0.16	0.3	0.27
Kidney				
Radiologic technologist	1.2	0.12	0.7	0.15
Radiologist	0.4	0.03	0.4	0.09
Other physician	0.2	0.02	0.3	0.06
Dentist	0.1	0.01	0.1	0.02
Dental hygienist	0.4	0.04	0.2	0.04
Nurse	0.7	0.06	0.6	0.12
Other	0.4	0.03	0.3	0.06
Liver				
Radiologic technologist	21.3	0.39	7.0	0.31
Radiologist	6.5	0.12	4.2	0.19
Other physician	3.0	0.06	3.0	0.14
Dentist	1.9	0.04	1.2	0.05
Dental hygienist	11.2	0.21	1.7	0.07
Nurse	12.9	0.24	5.6	0.25
Other	6.4	0.12	3.0	0.13
Lung				
Radiologic technologist	36.1	0.38	31.6	0.87
Radiologist	10.1	0.11	17.9	0.49
Other physician	5.3	0.06	13.1	0.36
Dentist	3.1	0.03	5.3	0.15
Dental hygienist	17.8	0.19	7.3	0.20
Nurse	20.9	0.22	24.8	0.68
Other	10.5	0.11	13.0	0.36
Oral cavity and pharynx				
Radiologic technologist	1.8	0.20	0.9	0.27
Radiologist	0.5	0.05	0.6	0.17
Other physician	0.2	0.03	0.4	0.11
Dentist	0.2	0.02	0.2	0.05
Dental hygienist	0.9	0.10	0.2	0.07
Nurse	1.0	0.11	0.8	0.23
Other	0.5	0.06	0.4	0.12
Ovary				
Radiologic technologist	–	–	1.4	0.20
Radiologist	–	–	0.9	0.13
Other physician	–	–	0.6	0.09
Dentist	–	–	0.3	0.04
Dental hygienist	–	–	0.4	0.05
Nurse	–	–	1.2	0.17
Other	–	–	0.7	0.09
Pancreas				
Radiologic technologist	4.6	0.31	3.0	0.22
Radiologist	1.3	0.09	1.9	0.14
Other physician	0.6	0.04	1.3	0.09
Dentist	0.4	0.03	0.5	0.04
Dental hygienist	2.9	0.20	0.7	0.05
Nurse	2.5	0.17	2.3	0.17
Other	1.3	0.09	1.3	0.10
Prostate				
Radiologic technologist	3.3	0.06	–	–
Radiologist	1.0	0.02	–	–
Other physician	0.4	0.01	–	–
Dentist	0.3	0.01	–	–
Dental hygienist	2.0	0.04	–	–
Nurse	1.8	0.04	–	–
Other	1.0	0.02	–	–
Red bone marrow				
Radiologic technologist	4.7	0.84	1.9	0.46
Radiologist	2.3	0.41	2.2	0.54
Other physician	0.9	0.16	0.9	0.22
Dentist	0.6	0.11	0.5	0.12
Dental hygienist	0.9	0.16	0.6	0.13
Nurse	2.6	0.46	1.9	0.46
Other	1.3	0.24	0.8	0.19
Stomach				
Radiologic technologist	40.0	0.39	25.1	0.50
Radiologist	12.1	0.12	15.2	0.30
Other physician	5.7	0.06	10.2	0.20
Dentist	3.6	0.03	4.4	0.09
Dental hygienist	19.4	0.19	6.0	0.12
Nurse	23.4	0.23	20.5	0.41
Other	11.9	0.12	10.7	0.21
Thyroid				
Radiologic technologist	39.8	2.23	222.0	2.57
Radiologist	16.1	0.91	163.0	1.92
Other physician	2.9	0.18	64.1	0.77
Dentist	4.1	0.23	35.3	0.42
Dental hygienist	19.7	1.11	63.6	0.74
Nurse	26.4	1.50	257.0	2.98
Other	14.6	0.82	123.0	1.42
Uterus				
Radiologic technologist	–	–	1.3	0.09
Radiologist	–	–	0.8	0.06
Other physician	–	–	0.2	0.02
Dentist	–	–	1.6	0.11
Dental hygienist	–	–	0.3	0.02
Nurse	–	–	1.1	0.07
Other	–	–	0.6	0.04
Remainder				
Radiologic technologist	41.7	0.84	18.9	0.39
Radiologist	12.6	0.25	11.7	0.25
Other physician	5.9	0.12	8.0	0.17
Dentist	3.7	0.08	3.3	0.07
Dental hygienist	22.8	0.46	4.5	0.09
Nurse	25.4	0.51	15.5	0.32
Other	12.3	0.25	8.4	0.18

*LAR* lifetime attributable risk (excess cases per 100,000), *LFR* lifetime fractional risk

The remainder included all other solid cancers, excluding non-melanoma skin cancer

## Discussion

This study presents data on potential cancer risks from occupational radiation exposure in a population of diagnostic medical radiation workers based on realistic exposure scenarios. The estimated risk was small in most cases but varied by sex and occupational group. Radiologic technologists had the highest LAR of cancer followed by nurses. Female workers had a higher projected radiation-related excess cancer risk than male workers, although women were exposed to lower radiation doses than men. Both LAR and LFR showed a tendency of temporal decrease with respected to year started work in all occupational groups. The LFR of thyroid and bladder cancer was high for both male and female radiologic technologists. These projections provide a quantification of potential cancer risk based on the current levels of occupational radiation exposure, and they help in identifying priority groups for radiation protection measures among medical radiation workers.

The projected cancer risk in medical radiation workers was lower than that estimated for staff working in cardiac catheterization in Italy [13] and vascular surgeons in Korea [14], although a direct comparison was not possible because of differences in the applied methods and exposure scenarios. A relatively low radiation exposure in our study group (i.e., the median effective dose was 0.78–22.86 mSv, in contrast to 46 mSv [interquartile range 24–64 mSv] for the Italian cardiac catheterization workers and 7.7 mSV among Korean vascular surgeons) would produce a smaller risk. The risk in this group would be even lower considering the tendency of decrease in the occupational dose by year of start of work. However, there was a wide variation in cancer risk by occupational group, and radiologic technologists and nurses who started work in 1991 had a non-negligible cancer risk, particularly female workers. Furthermore, median annual doses were used for estimating the risk in the RadRAT program because of the skewed distribution of radiation doses, and the estimated risk would be higher if mean doses were used. Some medical radiation workers tended not to wear dosimeters regularly [19], and therefore their actual dose levels would be higher than those reported. However, there is also a possibility of overestimation of risk, based on not taking into account possible reductions in occupational dose.

Female workers consistently showed a higher LAR of all radiation-related cancers combined for all occupational groups although these workers were exposed to lower doses of radiation than male workers [17, 27]. This result may be due to the combined effects of the higher radiosensitivity in women than in men (i.e., the higher coefficients of radiation dose in the excess risk model equations), younger age of first exposure in women, and higher baseline incidence rates for thyroid cancer in South Korea [28]. In addition, breast and thyroid cancer mainly contributed to this difference; thus, if both cancers were excluded, the LARs were found to be higher among males. Both the magnitude of baseline cancer risk and the level of radiation dose are important for a full assessment of establishing priorities for radiation protection. Although the level of exposure to radiation is one of the key components of the degree of cancer risk, it is necessary to incorporate the background risk of developing cancers and exposure information when assessing priority groups and designing interventions for radiation workers. These findings suggest that sex is an important determinant of occupational cancer risk, especially among the occupational groups with a high proportion of female workers, including medical radiation workers.

The difference in lifetime attributable cancer risk by occupational group may be attributable to differences in the distribution of age at first exposure and radiation doses between the groups. In this study, radiologic technologists and nurses had a higher cancer risk than other medical radiation workers of both sexes. The age of first employment for radiologic technologists and nurses was younger than that for other groups (e.g., the mean age was 22 years for female radiologic technologists and nurses, and 26 and 29 for radiologists and physicians, respectively), which contribute to the longer exposure duration. Furthermore, radiologic technologists had higher annual radiation doses than other groups and consequently have higher cumulative doses. In addition, the medical radiation workers in South Korea who were exposed to radiation earlier received higher doses of radiation [17, 29], which explains the observed tendency of temporal decrease in LAR considering the years of radiologic practice. This tendency was relatively smaller in radiologic technologists than in other groups, suggesting that excess lifetime risk in radiologic technologists was not decreased significantly by year of start of work, and therefore these professionals may have priority for radiation protection.

Among the cancer sites, the LFR of thyroid and bladder cancer in both men and women was higher than those of other organ sites. For thyroid cancer, high baseline incidence rate may contribute the high LAR and LFR. The thyroid incidence rate, used as the baseline incidence rate in the RadRAT program, was 52.7 per 100,000 for all ages and both sexes combined in 2010 based on the world standard population (http://www.ncc.re.kr), which was significantly higher than that found in other countries [30]. Compared to that of other cancer sites, a relatively high LAR of breast cancer among women and colon and stomach cancers among men may also be related to higher baseline incidence rates. However, the LFR of bladder cancer and leukemia was relatively higher than that of cancers in other organ sites due to the higher coefficients of radiation dose in risk model than other sites [15] and the low baseline incidences in South Korea [28]. However, further studies are warranted to explain this finding. The LAR of a few cancers (i.e., lung, stomach, bladder, and esophagus) was higher in men than in women but the LFR of these cancers was lower, probably because of higher radiation sensitivity in women and a relatively lower baseline incidence in women than in men.

This study has strengths. The first is the inclusion of all diagnostic medical radiation workers in South Korea between 1996 and 2011. Our analysis included all job categories, whereas previous studies limited the cancer risk analysis to specific procedures or occupational groups. In addition, individual organ doses were estimated on the basis of NDR data from 1996 to 2011. In contrast, previous studies obtained exposure information mainly from simulated measurements in short periods. Furthermore, the lifetime risk presented here was based on age- and sex-specific exposure scenarios separately for different occupational groups using data from a previous survey and a government-based centralized dosimetry registry. Our study population was more representative of the general population, and the exposure scenarios presented in this study were more realistic than those reported in previous occupational studies. Moreover, the enhanced projection models were applied to calculate the lifetime cancer risk from occupational radiation exposure.

There are some limitations to address. First, the dosimetry data may have underestimated the real exposure because not all workers wore badge dosimeters regularly [19]. Although KCDC continuously inspects unusual doses and corrects them, we acknowledge this limitation and the need to further evaluate the validity of badge doses. Reconstruction of historical badge doses for those who started work before 1996 is another potential uncertainty for risk estimation that needs to be improved. Second, the lifetime risk estimation of the model had uncertainties related to baseline incidence rates, changes in work practices, and survival rate because these variables may have changed by calendar year. These changes are inherent to risk projection studies. The assumed linear no-threshold risk model in the RadRAT may also be a subject of debate; however, this model was the most widely adopted and best-fitting model for explaining the radiation dose–response relationship based on current epidemiological data [31]*.* The estimated risks would be higher if no DDREF was applied. Our estimation program did not consider confounding factors or effect modifiers on cancer risk from radiation exposure, and many environmental and genetic factors may have modulated individual vulnerability to carcinogenic effects of radiation. Because the estimated risks reported here were based on scenarios rather than on individual exposures, the risk should not be interpreted as precise individual risk but can inform radiation protection policy at the population level.

## Conclusions

This study projected cancer risk from occupational exposure to radiation among medical radiation workers. The estimated risks for specific occupational groups, from highest to lowest, were generally in the order of radiologic technologists, nurses, radiologists, other physicians, dental hygienists, and dentists; however, the estimates for female radiologic technologists were considerably higher and warrant attention. Our findings provide an evidence of the magnitude of potential cancer risk considering the current levels of occupational radiation exposure. Our findings may also be used for establishing priorities for different populations, increasing awareness about the potential risk of radiation procedures, and making comparisons with other occupational and environmental risk factors. Lifetime risk estimation with individual exposure monitoring may help to timely assess the risk of cancer from radiation exposure and provide practical information for protecting medical radiation workers.

## Additional file

Additional file 1: **Figure S1.** Example of calculating excess lifetime risk for breast cancer. (DOCX 659 kb)

## Abbreviations

BEIR VII: Biological Effects of Ionizing Radiation VII
KCDC: Korea Center for Disease Control and Prevention
LAR: Lifetime attributable risk
LFR: Lifetime fractional risk
NDR: National Dosimetry Registry
UI: Uncertainty interval

## References

[1] UNSCEAR (2008). Effects of Ionizing Radiation: UNSCEAR 2006 Report to the General Assembly, with Scientific Annexes. Volume I. Effects of Ionizing Radiation.

[2] Yoshinaga S, Mabuchi K, Sigurdson AJ, Doody MM, Ron E (2004). Cancer risks among radiologists and radiologic technologists: review of epidemiologic studies. Radiology.

[3] Linet MS, Kim KP, Miller DL, Kleinerman RA, Simon SL, Berrington de Gonzalez A (2010). Historical review of occupational exposures and cancer risks in medical radiation workers. Radiat Res.

[4] U.S.EPA (2011). EPA Radiogenic Cancer Risk Models and Projections for the U.S. Population.

[5] ICRP (2007). The 2007 Recommendations of the International Commission on Radiological Protection. ICRP publication 103. Ann ICRP.

[6] Brenner DJ, Elliston CD, Hall EJ, Berdon WE (2001). Estimated risks of radiation-induced fatal cancer from pediatric CT. AJR Am J Roentgenol.

[7] Journy Neige M Y, Lee Choonsik, Harbron Richard W, McHugh Kieran, Pearce Mark S, Berrington de González Amy (2016). Projected cancer risks potentially related to past, current and future practices in paediatric CT in the United Kingdom, 1990–2020. British Journal of Cancer.

[8] Andersson M, Eckerman K, Mattsson S (2017). Lifetime attributable risk as an alternative to effective dose to describe the risk of cancer for patients in diagnostic and therapeutic nuclear medicine. Phys Med Biol.

[9] Land CE, Bouville A, Apostoaei I, Simon SL (2010). Projected lifetime cancer risks from exposure to regional radioactive fallout in the Marshall Islands. Health Phys.

[10] Hunter N, Muirhead CR, Bochicchio F, Haylock RG (2015). Calculation of lifetime lung cancer risks associated with radon exposure, based on various models and exposure scenarios. J Radiol Prot.

[11] Cardis E, Krewski D, Boniol M, Drozdovitch V, Darby SC, Gilbert ES, Akiba S, Benichou J, Ferlay J, Gandini S, Hill C, Howe G, Kesminiene A, Moser M, Sanchez M, Storm H, Voisin L, Boyle P (2006). Estimates of the cancer burden in Europe from radioactive fallout from the Chernobyl accident. Int J Cancer.

[12] Walsh L, Zhang W, Shore RE, Auvinen A, Laurier D, Wakeford R, Jacob P, Gent N, Anspaugh LR, Schüz J, Kesminiene A, van Deventer E, Tritscher A, del Rosarion Pérez M (2014). A framework for estimating radiation-related cancer risks in Japan from the 2011 Fukushima nuclear accident. Radiat Res.

[13] Venneri L, Rossi F, Botto N, Andreassi MG, Salcone N, Emad A (2009). Cancer risk from professional exposure in staff working in cardiac catheterization laboratory: insights from the National Research Council’s biological effects of ionizing radiation VII report. Am Heart J.

[14] Kim JB, Lee J, Park K (2017). Radiation hazards to vascular surgeon and scrub nurse in mobile fluoroscopy equipped hybrid vascular room. Ann Surg Treat Res.

[15] National Research Council, Division on Earth and Life Studies, Board on Radiation Effects Research, Committee to Assess Health Risks from Exposure to Low Levels of Ionizing Radiation (2006). Health Risks from Exposure to Low Levels of Ionizing Radiation: BEIR VII Phase 2.

[16] Korea Centers for Disease Control & Prevention. 2016 Report Occupational Radiation Exposure in Diagnostic Radiology in Korea. http://www.cdc.go.kr. Accessed 8 Nov 2018.

[17] Choi Y, Cha ES, Bang YJ, Ko S, Ha M, Lee WJ (2017). Estimation of organ doses among diagnostic medical radiation workers in South Korea. Radiat Prot Dosim.

[18] Choi Y, Kim J, Lee JJ, Jun JK, Lee WJ (2016). Reconstruction of radiation dose received by diagnostic radiologic technologists in Korea. J Prev Med Public Health.

[19] Korean Ministry of Food and Drug Safety (2013). Occupational Radiation Exposure and Health Effects in a Cohort of Diagnostic Radiation Workers in Korea.

[20] ICRP (1996). Conversion coefficients for use in radiological protection against external radiation. ICRP Publication 74. Ann ICRP.

[21] ICRP (2010). Conversion coefficients for radiological protection quantities for external radiation exposures. ICRP publication 116. Ann ICRP.

[22] Berrington de Gonzalez A, Iulian Apostoaei A, Veiga LHS, Rajaraman P, Thomas BA, Owen Hoffman F (2012). RadRAT: a radiation risk assessment tool for lifetime cancer risk projection. J Radiol Prot.

[23] Berrington de González A, Mahesh M, Kim K-P, Bhargavan M, Lewis R, Mettler F (2009). Projected cancer risks from computed tomographic scans performed in the United States in 2007. Arch Intern Med.

[24] Fresco R, Spera G, Meyer C, Cabral P, Mackey JR (2015). Imaging radiation doses and associated risks and benefits in subjects participating in breast cancer clinical trials. Oncologist.

[25] Hoang JK, Reiman RE, Nguyen GB, Januzis N, Chin BB, Lowry C (2015). Lifetime attributable risk of cancer from radiation exposure during parathyroid imaging: comparison of 4D CT and parathyroid scintigraphy. AJR Am J Roentgenol.

[26] Kellerer AM, Nekolla EA, Walsh L (2001). On the conversion of solid cancer excess relative risk into lifetime attributable risk. Radiat Environ Biophys.

[27] Lee WJ, Ha M, Hwang S-S, Lee K-M, Jin Y-W, Jeong M (2015). The radiologic technologists’ health study in South Korea: study design and baseline results. Int Arch Occup Environ Health.

[28] Jung KW, Won YJ, Kong HJ, Lee ES (2018). Community of Population-Based Regional Cancer Registries. Cancer statistics in Korea: incidence, mortality, survival, and prevalence in 2015. Cancer Res Treat.

[29] Lee WJ, Cha ES, Ha M, Jin YW, Hwang SS, Kong KA (2009). Occupational radiation doses among diagnostic radiation workers in South Korea, 1996-2006. Radiat Prot Dosim.

[30] International Agency for Research on Cancer. Global cancer observatory. World Health Organization. http://gco.iarc.fr. Accessed 8 Nov 2018.

[31] National Council on Radiation Protection and Measurements (NCRP) (2017). Implications of Recent Epidemiologic Studies for the Linear Nonthreshold Model and Radiation Protection.

