# Supplementary material for: Projected lifetime cancer risks from occupational radiation exposure among diagnostic medical radiation workers in South Korea
Source: BMC Cancer. 2018 Dec 4;18:1206. doi: 10.1186/s12885-018-5107-x (PMC6278159; doi:10.1186/s12885-018-5107-x)

Additional file 1: Figure S1. Example of calculating excess lifetime risk for breast cancer

<Example case>

Gender – female

Birth year – 1979

Job title – radiologic technologist

Exposure years – 2001-2039

Age at time of exposure – 22-60 years

Exposure scenario – occupational exposure each year for 39 years

Exposure Rate – chronic

Organ exposed – breast

Distribution of organ dose – lognormal probability distribution

Reference population – South Korea 2010

<Results>


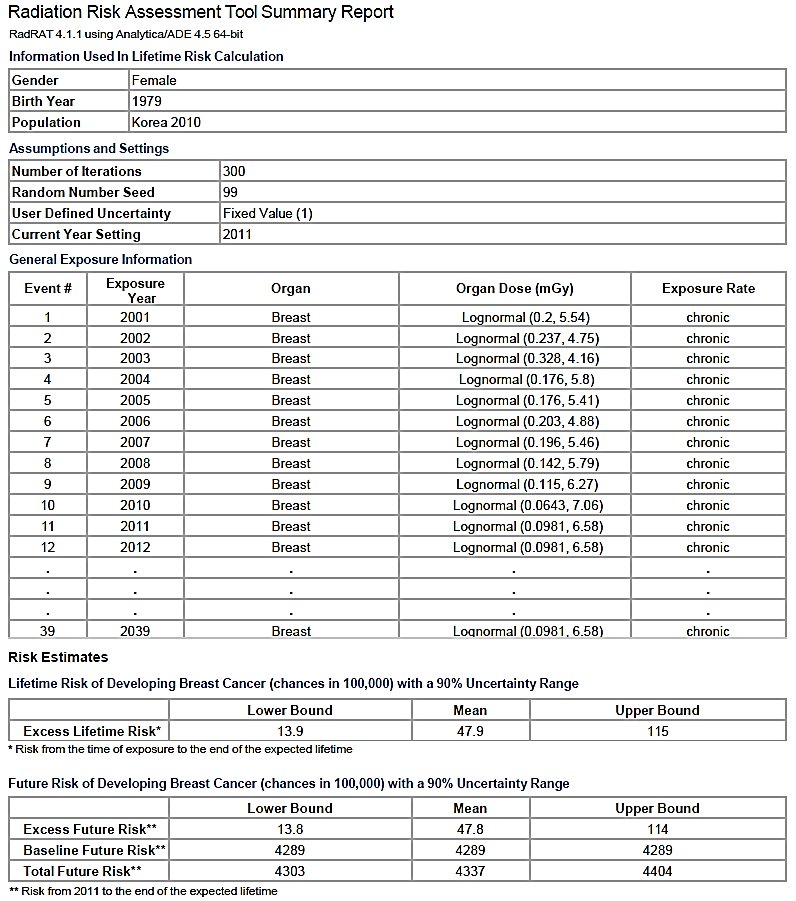

Supplement: Supplementary file 1 — Figure S1. Example of calculating excess lifetime risk for breast cancer. (DOCX 659 kb) [file 12885_2018_5107_MOESM1_ESM.docx]
